# Classical View on Nonclassical Crystal Growth in a Biological Setting

**DOI:** 10.1021/jacs.4c11940

**Published:** 2024-12-16

**Authors:** Richard Johannes Best, Deborah Stier, Lucas Kuhrts, Igor Zlotnikov

**Affiliations:** B CUBE - Center for Molecular Bioengineering, Technische Universität Dresden, 01307 Dresden, Germany

## Abstract

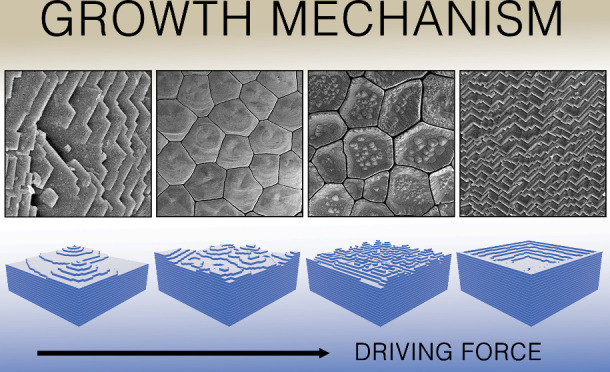

Crystallization by amorphous particle attachment, a nonclassical crystal growth mode, is prevalent in minerals formed by living tissues. It allows the organism to intervene at every step of crystal growth, i.e., particle formation, stabilization, accretion, and crystallization, and thus to orchestrate biomineral morphogenesis and crystallographic texturing; all toward achieving a required functionality for the organism. Therefore, significant effort is aimed at achieving similar control and crystal growth tunability through bioinspired and biomimetic synthetic means. This Perspective examines the driving forces and the kinetics of crystallization by amorphous particle attachment in a biological setting, and through an analogy to classical molecule-by-molecule crystallization, it establishes distinct crystal growth mechanisms. It underlines the role of physics and chemistry of materials in the “Growth and Form” of biogenic minerals.

## Introduction

The crystallization by particle attachment (CPA) phenomenon in geological and synthetic systems was first described in the 1960s, at a time when transmission electron microscopy became an essential tool in materials science and enabled such an observation.^1^ However, only in the last two decades has this so-called “nonclassical crystallization” route gained significant attention due to its prevalence in minerals formed by living organisms^2,3^ and owing to the novel notion that it can allow for more precise engineering of organic^4^ and inorganic particles.^5,6^ Today, abundant evidence from synthetic/geologic/biologic systems suggests that in contrast to a classical view that describes the process of crystal growth as a monomer-by-monomer addition,^7,8^ crystallization can proceed by accretion of higher order entities in the form of clustered ionic or molecular species, liquid droplets, and crystalline and amorphous particles.^2,9,10^

The first evidence for CPA in a biological setting, collected from larval spicules^11^ and spines of mature sea urchins^12^ demonstrated that these skeletal elements are formed through a transient amorphous phase. Further studies concurred that this special form of CPA, Crystallization by Amorphous Particle Attachment (CAPA), comprising several intermediate stages (an amorphous phase formation, aggregation, and only then crystallization) is a common biomineralization mechanism that is observed across phyla: in mammalian teeth,^13^ molluscan shells,^14−16^ corals,^17^ plants,^18^ and others.^3^

Significant effort has been directed at understanding the different aspects of biological CAPA by performing *in vivo*, *in vitro*, and synthetic studies, focusing on crystallization of calcium carbonates (CCs) and calcium phosphates (CPs), two of the most common minerals formed by organisms.^19^ To study the CAPA route, it is necessary to probe every intermediate crystallization step, each having its own unique thermodynamic and kinetic constraints. In the case of biogenic calcite, a polymorph of calcium carbonate (CaCO_3_), numerous studies provided insight into stabilization of the transient amorphous phase,^20,21^ solid-state transformation into the crystalline form,^22^ and other aspects, such as interaction with water^23^ and lattice strains and mosaicity.^24,25^ Furthermore, this work stimulated substantial research toward bioinspired and biomimetic CAPA routes of materials synthesis.^6,26,27^ However, a major aspect of this nonclassical crystal growth process remains elusive. Namely, we do not fully understand the forces that govern the growth of CAPA formed crystals in nature and eventually regulate their morphology.

## Crystallization in a Biological Setting

**Figure 1:**
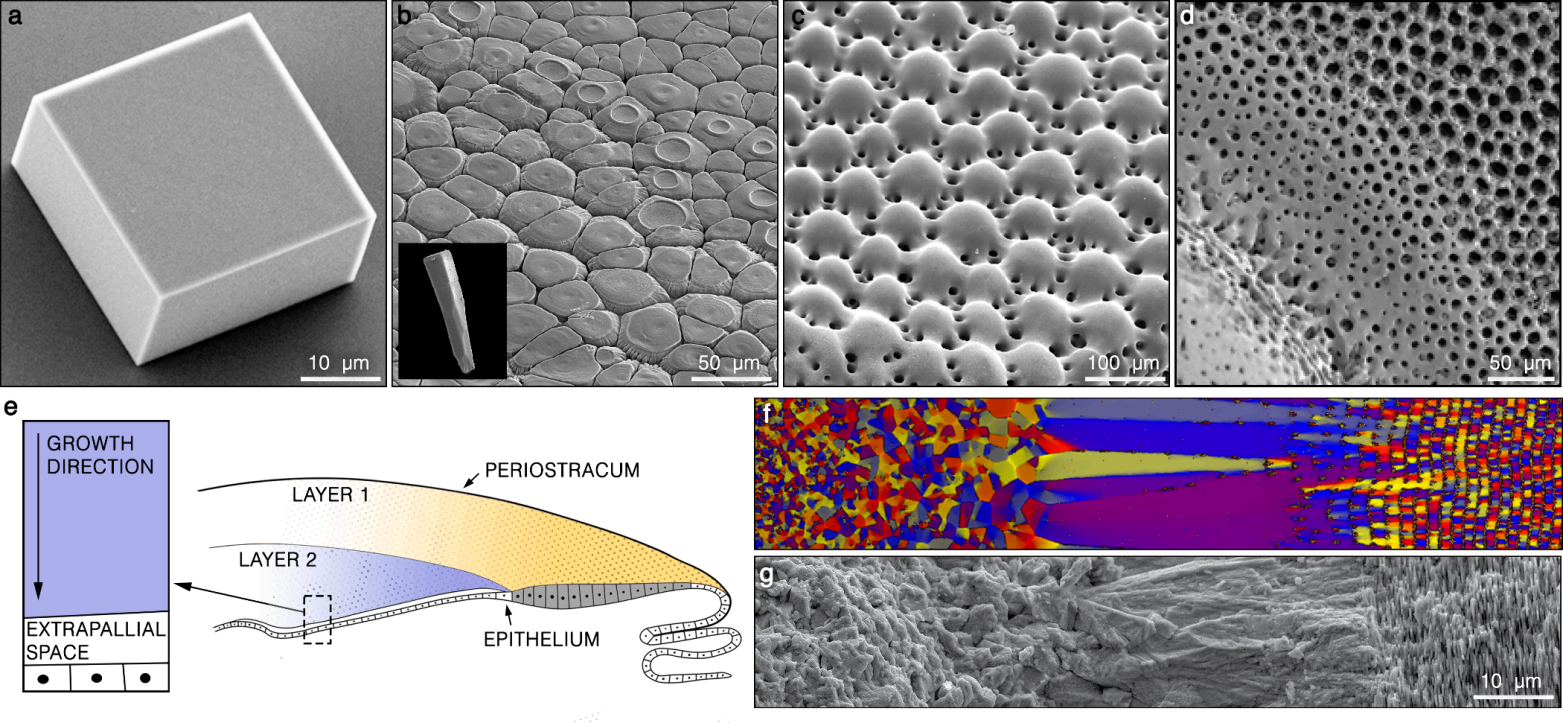
Crystallization by amorphous particle attachment in a biological setting. (a) Single crystal of classically crystallized calcite exhibiting a rhombohedral habit exposing the {104}-planes. (b) Prismatic ultrastructure in the shell of *Pinctada radiata*. The inset shows a prismatic single-crystalline unit. (c) Segment of the skeleton of a brittle star *Ophiocoma wendtii*. The entire structure is a single calcite crystal used for optical functions. Adapted from ref (32). Copyright 2003 American Association for the Advancement of Science. (d) Microstructure of tubercle in the skeleton of the sea urchin *Paracentrotus lividus*. Reproduced from ref (33). Copyright 2018 American Chemical Society. The entire structure is a calcite single crystal. Biominerals in panels b, c, and d were formed through an intermediate amorphous phase. (e) Schematic representation of a shell of a mollusc having two distinct ultrastructural layers. The two colors represent two individual ultrastructural layers sequentially formed on top of each other. (f) Phase-field simulation of an entire thickness of the shell of the cephalopod *Nautilus pompilius* having three ultrastructural layers made entirely of aragonite (from left to right: granular, prismatic, and nacre). The different colors represent different crystal orientations. For comparison, the actual microstructure of the shell is presented in panel g. Adapted from ref (44). Available under CC-BY 4.0. Copyright 2019 National Academy of Science.

Biomineralizing systems not only provide a unique opportunity to shed light on one of the most important questions in species evolution and other natural and earth sciences but also have the potential to inspire and provide explicit toolkits for engineering and design of new materials.^5,26,28,29^ Mineral crystals formed by living organisms through the CAPA route exhibit lattice properties and complex morphologies that contradict the thermodynamically stable habits expected from their atomic structure. In the case of calcium carbonate, for example, the {104}-planes of the hexagonal lattice define the most stable rhombohedral habit of calcite (Figure 1a).^30^ In contrast, organisms are known to form single crystals of calcite with various shapes, having not a single {104}-plane exposed: from columns in molluscan shells (Figure 1b)^31^ and spherical lenses in brittle stars (Figure 1c)^32^ to hierarchical porous microstructures in sea urchins (Figure 1d).^33^ At the same time, while still forming extremely complex morphologies, biogenic calcite crystals formed via the ion-by-ion route, such as those covering coccolithophore cells, exhibit clear facets belonging to the {104} family.^34,35^

Still, a biological environment is probably the most complex setting for studying the fundamentals of crystallization and understanding the energetic landscape that drives CAPA. Here, cellular and genetic regulation is postulated to have the utmost control over every aspect of mineral formation and morphogenesis. From a widely accepted biological point of view, biomineralized tissues, like any other tissue, evolved under certain functional constraints and adapted to perform a specific task for the organism. For example, focusing on biogenic calcite, the emergence of complex shell architectures, like the crossed-foliated ultrastructure in the shell of the limpet *Patella vulgata*, was assigned to patterning by the mantle tissue,^36^ and the crystallographic texture in the prisms of *Pinctada* bivalves was associated with mechanically driven functionality.^37,38^ Other works attempt to identify an explicit macromolecular framework that is responsible for the morphogenesis of various mineral morphologies and the assemblies they form.^39−41^ Some of these seminal works provided significant insight into possible mechanisms for mineral–organic interactions and inspired multiple studies on crystallization in the presence of various organic and inorganic impurities. However, we still do not understand how nature takes advantage of thermodynamic and kinetic principles to generate complex morphologies and what the interplay is between the physics of materials and cellular control in this process.

In the past decade, it was demonstrated that at least in the case of molluscan shell mineralization, which proceeds in an extracellular space between the mantle tissue and the growing front of the shell, the control of all CAPA steps may occur indirectly.^42−51^ During shell development, a purely organic periostracum is formed first (Figure 1e). Then, shell ultrastructures are deposited in an extrapallial space with the epithelial cells of the mantle providing all the necessary organic and inorganic precursors for the different structures to form. These are arranged in layers that lay parallel to the outer surface of the shell, each layer exhibiting a unique assembly of single-crystalline mineral units (platelets, prisms, needles, laths) attached together by an organic membrane.^43^ It was postulated that the cellular component generates all the necessary physicochemical boundary conditions for mineral formation and morphogenesis to emerge through self-assembly. In essence, it was hypothesized that the cells form a microenvironment that promotes spontaneous mineral crystallization via the CAPA route. Here, genetic control is exerted by generating the needed physical conditions, such as pH and temperature, and by secreting the necessary inorganic and macromolecular precursors, such as proteins and polysaccharides, which are responsible for calcium carbonate polymorph selections, crystal nucleation, growth, and habit modification and for regulating the kinetics of every step of the crystallization process. One may say that the proteins are exerting passive control as a solute rather than orchestrating every single event during molluscan shell formation by biochemical means directly.

Starting with a first paper in 2014^42^ that showed that the formation of the prismatic ultrastructure in the shell of *Pinna nobilis* follows all classical predictions for crystal growth and coarsening, we continued to validate that concepts from physics of materials and soft matter physics may account for various structural and crystallographic characteristics of other molluscan shell ultrastructural motifs.^43−48^ This controversial idea has a number of significant implications. First, it suggests that crystal lattice complexities observed in biominerals formed through the CAPA route are merely a consequence of the growth process and not a result of guided adaptation toward a specific functionality.^24,25^ Second, if basic principles borrowed from physics of materials can account for the morphogenesis of biomineralized entities, we can potentially apply classical computational and numerical methods to describe their growth analytically, in time, and in space. Indeed, in recent years, a number of analytical and computational approaches, such as the grain growth and coarsening formalism and the phase-field approach were successfully applied to reproduce the intricate structural motifs observed in molluscan shells and corals (Figure 1f,g).^45,47,49−51^

Taking this point of view into consideration, biogenic minerals, focusing on the nonclassically formed biogenic calcite comprising the various molluscan ultrastructures, are an ideal system to understand the fundamentals of CAPA. Coincidentally, for almost two centuries, molluscan shells have also been an exemplar model to study the process of biomineralization^52^ and, due to their superior mechanical properties, are one of the most synthetically mimicked and mechanically characterized biological materials.^53^ So, can we identify explicit CAPA growth mechanisms in this biological setting and understand how these relate to monomer-by-monomer processes described by the classical crystal growth formalism?

## Classical Mechanisms of Crystal Growth

**Figure 2:**
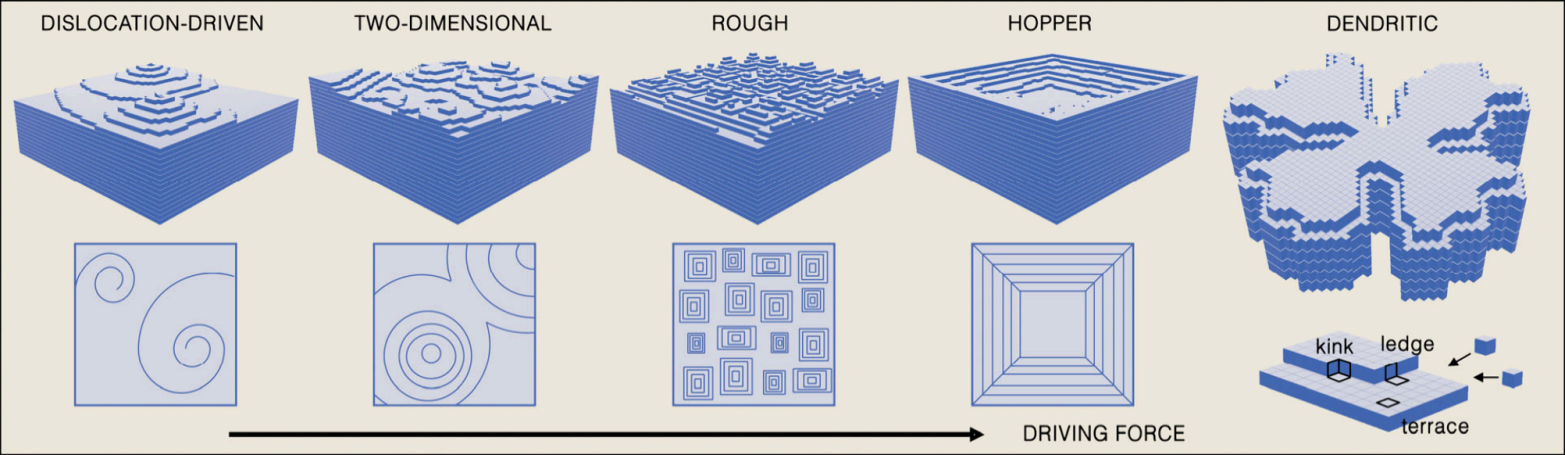
Schematic representation of classical crystal growth mechanisms. Upper row: a 3D topography of a growing crystal front as a function of an increasing driving force resulting in five distinct growth mechanisms (from left to right): screw dislocation assisted growth, two-dimensional growth, rough growth, hopper growth, and dendritic growth. Lower row: a 2D projection of the respective crystal growth fronts. The incorporated crystal components are represented by the cubic units that are incorporated at one of three possible locations: kink, ledge, or terrace. Note that during dendritic growth mode, the cubic crystal habit is lost to accommodate the dendritic crystal morphology while maintaining the periodic organization of the cubic building blocks.

According to our fundamental understanding of the classical crystallization processes, the growth mode of a single crystal strongly depends on the magnitude of the force that drives the process of its nucleation and subsequent growth. In the case of supersaturation driven growth, after nucleation, five crystal growth mechanisms are commonly distinguished (Figure 2). Adapting the terrace–ledge–kink (TLK) model of crystal growth developed during the early- and mid-1900s,^54,55^ these growth modes can be described by an interplay between the ability of individual entities to arrive to the front of the growing crystal and to be incorporated into it.^56,57^ Another way of looking at this classical description of crystal growth is asking the question of how easy it is for a crystal surface to grow under the given conditions?

Incorporation of a new lattice unit at a kink site is energetically the most favorable scenario. In the case of screw-dislocation-driven growth, sometimes referred to as spiral growth, the core of a dislocation provides a kink on an otherwise flat surface. At a low driving force for crystal growth, i.e., low supersaturation, these sites serve as nucleation centers for new crystal layers. As one can expect, in this case, the process of crystal growth is slow, as the incoming crystal entities need to reach the kink via diffusion on the crystal surface or in the bulk of the solution. In fact, in a sense, this process is limited by both diffusion and incorporation. After reaching a certain supersaturation—a higher driving force, nucleation of a new layer on a flat surface of the crystal, i.e., on a terrace, becomes energetically plausible. As a result, two-dimensional islands are formed and spread to generate a new crystal layer. Therefore, this mode of growth is called two-dimensional or birth and spread growth. In both mechanisms described above, growth proceeds by incorporating new crystal units at ledge (step) sites, and a new smooth surface is formed. At even higher driving forces, incorporation site discrimination is lost, and the surface of the crystal becomes rough. Here, the new lattice units are adsorbed immediately after reaching the surface, and the growth is relatively fast.

A special case of growth at high supersaturation levels is the hopper growth mode. Here, the edges of the crystal grow faster than the facets, forming a morphology reminiscent of a hopper container. This morphology is regularly found in bismuth, quartz, gold, halite and calcite.^58,59^ The exact mechanism of hopper crystal formation is not fully understood. However, it is well established that it emerges at high driving forces with limited mobility of the precursors and is therefore a diffusion limited phenomenon. Some even cite the so-called Berg effect,^60^ which assumes higher supersaturation levels at the edges of a growing crystal than at its center. Again, at low precursor mobility, the high driving force will result in faster growth of the edges compared to crystal facets and the emergence of a hopper morphology.^61^ Finally, at very high supersaturation levels (high driving force), a rapid development of dendritic crystal morphology is observed. Intuitively, under these conditions, a configuration that has a large surface area is favorable. The crystal loses its initial habit to accommodate kinetically uninhibited incorporation of new arriving entities forming a branched crystal morphology.^62,63^ It is important to note that a dendrite is a form that still exhibits single crystalline properties.

These five mechanisms are central to our understanding of synthetically and geologically formed organic and inorganic materials and play a key role in the design and engineering of single crystals across scales, from nanometers to meters.^64^ They will also serve as the basis for discussing growth by the CAPA mechanism.

## Mechanisms of Crystal Growth by Amorphous Particle Attachment (CAPA)

**Figure 3:**
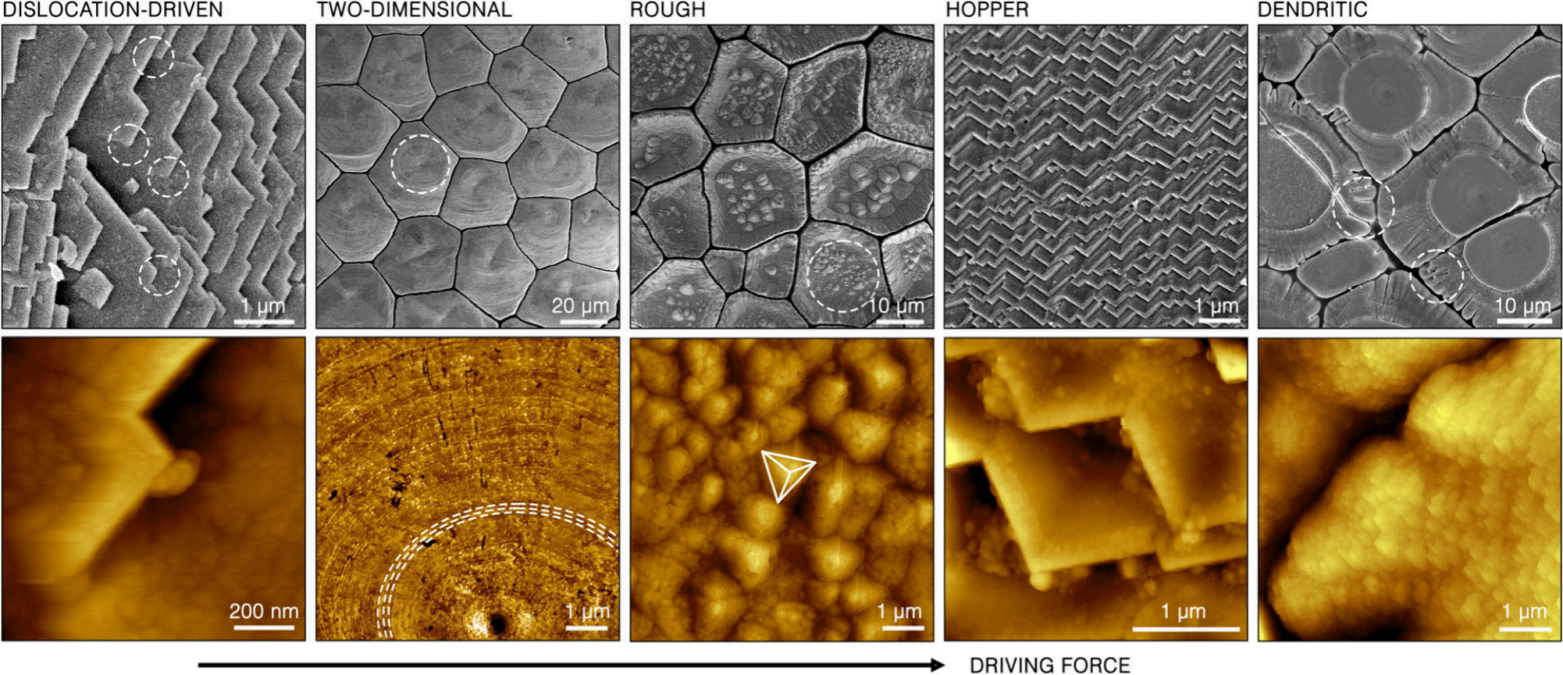
Biogenic crystals of calcite formed through particle attachment. Data for each growth mechanism presented in columns. Upper row: scanning electron microscopy images of five distinct growth mechanisms (from left to right): screw dislocation assisted growth, two-dimensional growth, rough growth, hopper growth, and dendritic growth, found in the crossed-foliated ultrastructure in the shell of *P. vulgata*, the prismatic ultrastructure in the shell of *P. nigra*, the prismatic ultrastructure in the shell of *P. nobilis*, the crossed-foliated ultrastructure in *P. vulgata*, and the prismatic ultrastructure in the shell of *P. radiata*, respectively. Bottom row: atomic force microscopy images of magnified particle accretion patterns corresponding to features marked in electron microscopy images in the upper row by a white dashed line, respectively. The dashed lines in the two-dimensional accretion image trace the extending front of the flat surface. The pyramid in the rough accretion image underlines the exposed {104} planes of calcite. The images of *P. nigra* and *P. nobilis* were adapted with permission from ref (25). Copyright 2021 John Wiley and Sons.

Although the particulate substructure in biogenic calcite and aragonite was previously recorded and analyzed in numerous molluscan species,^3,65,66^ only in 2021 were particle arrangement patterns used to identify and discuss accretion kinetics during crystal growth.^25^ By looking at the initial stages of prismatic ultrastructure formation in the shells of *Pinctada nigra* and *Pinna nobilis*, it was possible to describe accretion patterns analogous to two-dimensional and rough growth mechanisms, respectively (Figure 3). These patterns were revealed by bleaching the prismatic ultrastructure in 5 wt % of sodium hypochlorite, a process that removed the organic phase and loosely associated particles. In the case of two-dimensional growth, a clear nucleation center and circular arrangement of approximately 70 nm particles was observed. As predicted by the nonclassical growth formulation, this growth mode generated a relatively flat surface. It is important to note that a similar growth mechanism was previously observed during growth and crystallization of a synthetically prepared amorphous calcium carbonate (ACC).^67^ In contrast, the rough growth mode produced multiple triangular pyramids on the growth front, exposing the {104} family of crystallographic planes of calcite. Also here, the sizes of individual particles are in the 70 nm range. Similar substructure was, for example, previously observed in calcitic sponge spicules.^68^ A dendritic growth mode can also be easily identified in many structures in many species. Here, we show an example of a dendritic morphology and particulate substructure again using a calcitic prismatic ultrastructure in the shell of *Pinctada radiata* (Figure 3).

Having identified three out of five classical crystal growth mechanisms in molluscan minerals, we are left with dislocation driven and hopper crystal growth modes. Curiously, as mentioned above, calcite is one of the materials known to form hopper crystal morphology in geological and synthetic systems, but it has not been reported as such in a biological one.^69^

**Figure 4:**
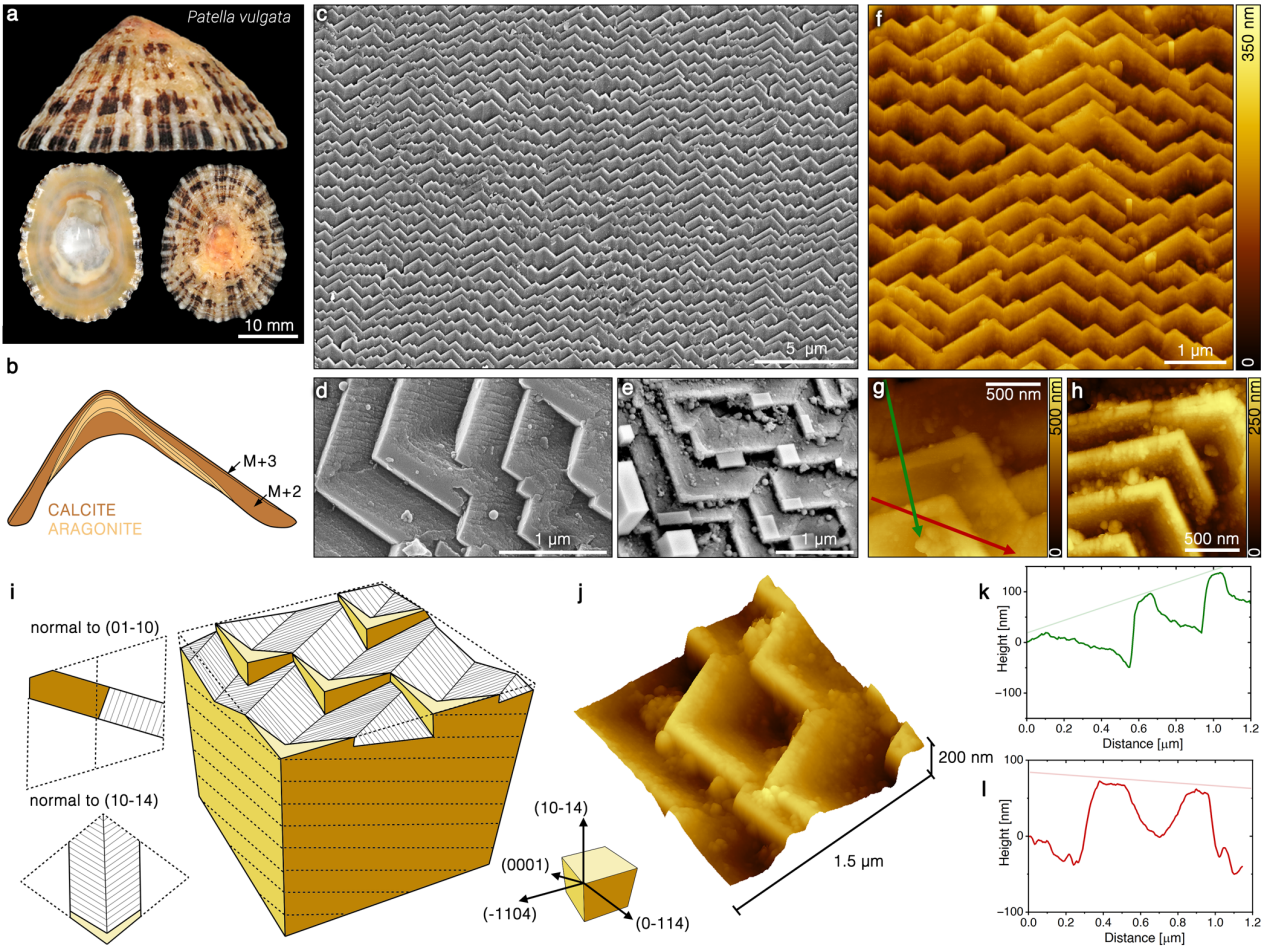
Foliated ultrastructure as a skeletal hopper crystal. (a) Shell of *P. vulgata*. (b) Schematic section of the shell from the upper panel in which the layers containing the foliated and the cross-foliated ultrastructures (M+3 and M+2, respectively) are labeled. (c) Scanning electron microscopy image of the growth front of the M+2 layer showing the surface of the crossed-foliated (CF) structure. (d) High-magnification electron microscopy image of a tilted foliated structure. (e) Electron microscopy image of the foliated structure after a calcite overgrowth experiment in vitro. The rhombohedral patches on top of the laths are calcite crystals formed as the result of the experiment. (f) Atomic force microscopy image of the growth front of the M+2 layer showing the topography of the foliated structure. (g) High-magnification atomic force microscopy image of the growth front of the M+2 layer. The red and the green arrows mark the location of the height profiles shown in panels k and l, respectively. (h) High-magnification atomic force microscopy image of the growth front of the M+2 layer. The background of the image was subtracted to demonstrate that the leading {104}-facets of the laths lay on the same plane. (i) Schematic representation of the foliated structure showing the crystallographic and morphological characteristics of an individual lath and the entire ultrastructure. The colored facets represent the equivalent {104}-planes of the calcitic lattice. (j) Three-dimensional rendering of the surface of the growth front of the M+2 layer taken using an atomic force microscope. (k, l) Height profiles across the laths following the green and red arrows in panel g, respectively.

Two of the most common calcitic molluscan shell ultrastructures are the foliated and crossed-foliated architectures comprising, for example, the outer layer of the shell of the limpet *Patella vulgata* (Figure 4a,b). The basic building units of these ultrastructures are calcitic narrow laths that are assembled side to side to form a calcitic sheet (foil). These sheets are stacked on top of each other to form a crystallographically coherent domain (Figure 4c,f). In the case in which the crystallographic misorientation between stacked domains is irregular, the ultrastructure is called foliated, whereas when these domains are stacked together to form another hierarchical level, namely, sequential switching between two specifically oriented domains, the ultrastructure is called crossed-foliated. In *P. vulgata*, both are present and are called M+3 and M+2 layers, respectively (Figure 4b).^36^ Focusing on the microstructure of these domains, the growth front of each (Figure 4c–h) clearly shows the layered architecture made of laths that emerge to the surface as sharpflat pyramids with two well developed sides and one upper undeveloped leading side, all belonging to the {104}-plane family (Figure 4i).^36^ This crystallography was further demonstrated by an overgrowth experiment. After keeping a piece of the shell in an oversaturated calcium carbonate solution, classical rhombohedral calcite nanoparticles formed epitaxially on these three facets (Figure 4e).

In fact, the upper face of the lath demonstrates a typical hopper morphology where the edges connecting the {104}-planes grow faster than the planes, thus leaving a dip behind (Figure 4j). Astonishingly, looking at the height profiles across a number of laths (Figure 4k,l), it becomes evident that, all together, the leading {104}-faces of the different laths in a domain form a pseudo-{104} plane. With a certain approximation, the entire foliated domain is a perfect skeletal hopper crystal where a number of single hopper units are interconnected to form a continuous single crystalline array.^58^

After the existence of the fourth crystal growth mechanism was demonstrated in the foliated ultrastructure of the shell of *P. vulgata*, it is relatively simple to find the final screw-dislocation-mediated growth mode. As mentioned above, hopper morphology arises under conditions where the edges of the crystal grow faster than the facets due to a significant reduction in the force driving the crystallization process. Therefore, at the facets, the growth is expected to proceed following a mechanism that requires a lower driving force, such as screw-dislocation-mediated mode. Indeed, looking at the upper faces of the laths, numerous screw dislocation sites are evident (Figure 3). While the growth of a single foliated domain in length proceeds by lath elongation, its growth in thickness, i.e., the formation of new foils, occurs with the help of screw dislocations.

This analysis demonstrates stark similarities in crystallization growth mechanisms between the classical and the nonclassical crystallization processes. However, whereas the driving force for classical crystallization can be estimated by the level of precursor supersaturation, the force driving the process of nonclassical crystallization, CAPA in particular, requires further discussion.

## Driving Force and Kinetics of Crystallization by Amorphous Particle Attachment

The most obvious hypothesis, after drawing an analogy to monomer-by-monomer growth, is that if classical growth is driven by supersaturation, then growth mechanisms of CAPA are correlated with amorphous particle concentration in the extrapallial space. Indeed, it is well established that the growth kinetics of colloidal crystals depends on the concentration of particles in the crystallizing medium.^70^ Furthermore, besides the hopper growth mechanism, all classically described growth modes were previously demonstrated in various synthetic colloidal systems, including synthetic CAPA of calcite.^67,71,72^ However, a direct correlation between particle concentration and a specific growth mode as described by classical crystallization (Figure 2) was not established, mainly because they could not be observed in a single system. Furthermore, whereas the kinetics of particle aggregation in colloidal suspensions is also strongly influenced by particle concentration, here too, its relationship to a specific growth mode cannot be established, simply because the aggregates have no crystalline order or TLK sites. All in all, one thing is clear, classical particle aggregation and colloidal crystallization are faster at a higher level of precursor particle concentration. However, unlike classical crystallization where supersaturation is an explicit term in the calculation of the change of Gibbs free energy upon crystallization,^73^ concentration in the case of CPA has no obvious thermodynamic influence on crystal growth as a driving force.

Particle accretion in colloidal suspensions is usually described within the thermodynamic framework of the Derjaguin, Landau, Verwey, and Overbeek (DLVO) theory and its adaptations.^74,75^ Here, the interaction between particles in a solution is defined by an interplay between attractive van der Waals forces and electrostatic surface charge repulsion. Its simplest form predicts that below a critical distance between two colliding particles, the entities will aggregate and remain as such due to an energy trap formed by the van der Waals force. This process has a statistical nature that is described by the collision efficiency and collision frequency. The former describes which portion of collisions results in a successful attachment, and the latter describes how often the particles actually collide. Whereas collision efficiency carries some physicochemical characteristics, collision frequency mostly depends on the concentration of particles in a solution. In essence, with a higher particle concentration, intimate interparticle interactions are more frequent, and even at a low thermodynamic driving force, aggregation may proceed just because the particles are being persistent. In addition to van der Waals, other possible thermodynamic driving forces are the reduction of surface energy upon accretion, various solvent effects, hydrophobic interactions, and external forces.

Heterogeneous CAPA in molluscs is not a standard DLVO setting for many reasons. First, the extrapallial space where the growth front evolves is reported to be as thin as 100 nm.^76^ Regardless whether the ACC particles form in that space or are secreted into it by the mantle cells, there is simply not enough room for a significant interaction between particles,^77^ having a diameter at the 70 nm range, before they reach the growth front. This is clearly demonstrated by the second point, namely, by the fact that aggregation of ACC particles in the extrapallial space was never previously reported and crystallization seemingly occurs only heterogeneously on the forming biomineral unit. Here, the large interaction cross-section of ACC particles with the CC surface is responsible for the preferential ACC–CC over ACC–ACC particle association. Finally, as in all the provided examples (Figure 3), the growth front exhibits crystallographic characteristics of calcite and well-defined TLK sites, it appears that ACC particles crystallize upon attachment. Nevertheless, it is still possible to employ notions from DLVO theory to describe CAPA in this biological setting.

The organisms orchestrate every step of the CAPA process. As was demonstrated,^42−51^ in addition to biochemically mediated mineral precursor transport, all aspects of ACC particle formation, stabilization, and crystallization are most probably regulated by setting the physicochemical boundary conditions driving calcification and supervising its kinetics. The actual thermodynamic drive for ACC–CC particle to surface attachment and the crystallization process can be evaluated within the DLVO framework, which would most probably include forces stemming from van der Waals interactions, steric and depletion forces, reduction of surface energy,^78^ entropy gain due to surface dehydration,^79^ and ACC to CC transformation. Therefore, similarly to the case of classical crystal growth, TLK site selectivity is driven energetically by forces described by the DLVO theory, i.e., at lower driving forces, a site with a higher reduction in free energy is preferred.

In essence, the supersaturation-based free energy gain in the classical context can be replaced by DLVO forces in the CAPA model. The magnitudes of these entropic and enthalpic contributions can be relatively easily controlled by the organism by tuning the chemistry of the extrapallial liquid and auspiciously experimentally determined. For example, by adjusting the depletant composition or the ionic strength of the extrapallial space, mollusks can attain an utmost control of ACC particle interactions, aggregate nucleation, and assembly.^80,81^ In contrast, collision frequency, which scales with the concentration of ACC particles in the extrapallial space, is not affected by nor does it have an influence on the actual driving force and will mainly determine the speed of the process.

We established that interactions described by extended DLVO theory can define the driving force for CAPA and determine the energetic landscape for selective particle attachment. Collision frequency, i.e., particle concentration, determines the time scale of the growth process. The remaining question is whether precursor particle concentration can also discriminate a specific crystal growth mechanism (Figure 3). As mentioned before, CAPA in molluscs is a special case, where the motion of ACC particles is restricted by an extremely thin extrapallial space. In fact, they can move in only two dimensions just above the crystallizing surface. We postulate that at low concentrations, the particles will migrate above the surface practically undisturbed by other particles in the medium until a preferable attachment site is reached (dislocation-driven and 2D growth). Increasing the concentration will significantly affect their ability to move above the surface and reach a preferred TLK site. In combination with increased particle–surface collision frequency, a rough growth or a hopper mechanism will be established. We further postulate that at very high concentrations, the particles will crowd the extrapallial space, and dendritic growth will emerge. Therefore, unlike supersaturation in the classical case, at a given driving force, precursor particle concentration will determine CAPA growth mechanism kinetically.

## Morphogenesis in a Biological Setting

The notion that growth mechanisms of crystals formed via the CAPA route, at least in the molluscan setting, adhere to the classical description of crystallization must be considered when analyzing the process of their morphogenesis and crystallographic texture formation. We established that just like supersaturation in the classical case, in addition to other thermodynamic considerations, ACC particle concentration drives the growth of calcite crystals (Figure 3). Therefore, it is reasonable to assume that the formed crystals also exhibit crystallographic properties of an analogue formed molecule-by-molecule. Indeed, synthetic calcite crystals produced via the CAPA route exhibit the classical rhombohedral habit and its modifications, depending on the added impurities.^82^ However, in molluscan shells, the calcitic mineral units do not display such characteristics (Figure 3).

As was shown in recent publications,^42−51^ there are two main forces that define the morphology of CAPA-formed calcite building units in molluscan shells: competition for space and the orientation of the initial nucleus. CAPA in molluscs is a unique case, where, unlike crystallization in bulk, the crystals form on an organic template and evolve unidirectionally, toward the inner part of the shell (Figure 1).^45^ As demonstrated in the case of the prismatic ultrastructure (Figure 3), the shape of the minerals in the direction perpendicular to their growth shows the classical space-filling Voronoi construction morphology, and its change with the direction of growth follows predictions of grain growth and coarsening formulated for classical material systems.^31,83^ However, their growth in length is unconstrained; therefore, the growth front, similar to that observed in the foliated ultrastructure (Figure 3), is free to develop clear crystallographic features.

Finally, the crystallographic orientation of the nucleus with respect to the direction of unidirectional growth is pivotal in determining the ability of the mineral units to mature and to fill that space. The fact that ACC particles crystallize immediately after reaching their final location enforces crystallographic constraints on the growth kinetics that adhere to the mechanisms discussed in this work (Figure 3). As an example, the foliated structure will grow faster in the direction of the *c*-axis of calcite owing to a simultaneous fast hopper growth mode of all {104}-planes at the front of the laths (Figure 4). However, it will grow much slower in the direction perpendicular to the lath surface as this growth proceeds by the slow screw-dislocation-mediated mechanism.

It is important to point out that according to our observations, the kinetics of ACC to CC phase transformation after aggregation plays a significant role in the ability of the organism to manipulate and shape the morphology of the CAPA-formed crystals. For example, it is safe to assume that, unlike biogenic calcite in molluscs, phase transformation in brittle star lenses was delayed, allowing for the development of spherical shapes having no obvious crystallographic characteristics of calcite (Figure 1c). Most probably, this is achieved through an increased level of magnesium impurities well-known to delay ACC crystallization.^20,84^

## Conclusions and Outlook

Using analogy to classical crystal growth, we describe the kinetics and thermodynamics of crystallization by amorphous particle attachment (CAPA) in a biological setting and correlate it with the concentration of amorphous particles in the crystallizing medium. All five classical crystal growth mechanisms are demonstrated in calcitic building blocks of the shells of a number of molluscs formed through an intermediate amorphous calcium carbonate phase. Despite being formed under biological control, together with our recent work, this perspective underlines the role of physics and chemistry of materials in the “Growth and Form”^85^ of biogenic minerals. Genetic regulation exerts itself through strict control of the microenvironment, in which spontaneous self-assembly and crystallization occur.

Classical crystallization processes can be quantitatively described by a number of analytical models that take into account parameters describing the crystallization driving force, nucleation mode, and crystal growth kinetics. Due to the multitude of chemical and physical parameters that govern every step of CAPA, a quantitative understanding of this nonclassical crystallization process remains elusive. Obtaining a comprehensive analytical description of crystallization by amorphous particle attachment is crucial for both the study biomineral morphogenesis and the design of novel materials employing CAPA. This is most vividly demonstrated by the case of calcitic brittle star lenses, where we postulate that an increase in the energetic barrier of the crystallization step and/or a decrease in the barrier of the particle aggregation step allows for the formation of spherical calcite morphologies. We believe that future work should focus on isolating and carefully probing every step of CAPA by synthetic means, bringing us closer to the understanding the phenomenon semiempirically.

An important aspect of the CAPA process not covered in this text is the emergence of the crystallographic texture. Whereas biogenic calcite crystals exhibit single crystalline properties, recent work suggests that their unique formation process has a direct and also a spontaneous effect on local crystal lattice properties, distortions, and rotation.^24,25^ We postulate that an interplay between the morphogenic forces and crystallographic constraints hypothesized here is at the heart of biomineralization across scales, from crystallization of a single microscopic building block to the emergence of the shape of the entire tissue.
